# Telegraph Cable in Sewers

**Published:** 1881-07

**Authors:** 


### TELEGRAPH CABLE IN SEWERS.

An important experiment looking to the disuse of telegraph poles in cities is being made in Washington, D. C., by the Mutual Union Telegraph Company. Having received permission to run their wires through the common sewers of the city the company began the work of placing the wires June 6. The wires which are needed for the city service and for connection with lines outside the city are twisted cable form and covered with a non-conductor and waterproof coating. Outside the city limits these wires emerge from the sewers and join those placed upon poles. The cable made of the twisted wires is attached firmly to the arched roof or top of the sewer, and thus raised above all interference from water, except in case of floods. The cables are laid by men in rubber clothing and provided with safety lanterns, provision being made for conducting fresh air to the workmen by means of India rubber tubes attached to their suits. The wires are passed down through the man holes of the sewers.—*Scientific American.*

